# Identifying Candidate Genes for Cotton Fruit Branch Length Using BSA-Seq and RNA-Seq

**DOI:** 10.3390/plants15081192

**Published:** 2026-04-13

**Authors:** Penglong Wang, Yanlong Yang, Guoyong Fu, Yang Jiao, Zhenzhen Wang, Jun Ma, Chengxia Lai, Chunping Li, Haijiang Xu, Yunlong Zhai

**Affiliations:** 1 College of Agriculture, Tarim University, Alar 843300, China; plwang2025@163.com; 2 Xinjiang Key Laboratory of Cotton Genetic Improvement and Intelligent Production, Xinjiang Cotton Technology Innovation Center, Cotton Research Institute of Academy of Agricultural Sciences of Xinjiang Uyghur Autonomous Region (National Cotton Engineering Technology Research Center), Urumqi 830091, China; yangyl0629@163.com (Y.Y.); fgyong1212@xaas.ac.cn (G.F.); jycotton@163.com (Y.J.); shenhuawzz@163.com (Z.W.); xj.majun@163.com (J.M.); chunpin96@126.com (C.L.)

**Keywords:** cotton, fruit branch length, BSA-Seq, RNA-Seq, candidate genes

## Abstract

Fruit branch length in cotton is a key trait influencing plant architecture and suitability for mechanisation; elucidating its molecular regulatory mechanisms is crucial for breeding varieties with desirable plant architecture. In this study, an F_2_ segregating population was established using the long-fruit-branch upland cotton line L16 and the short-fruit-branch line S14 as parents. By integrating morphological, cytological, and omics approaches, we systematically analysed the underlying mechanisms of variation in fruit branch length. Phenotypic analysis indicated that the inter-node elongation rate of the first fruit branch in L16 was significantly higher than that in S14. Tissue section observations revealed that the length of cortical parenchyma cells in L16 was significantly greater than that in S14, suggesting that the difference in fruit branch length primarily stems from variations in the extent of cortical parenchyma cell elongation. BSA-Seq analysis identified five QTL regions significantly associated with fruit branch length, encompassing 82 coding genes. Further RNA-Seq analysis of the fruit branch initiation stage (T0) and rapid elongation stage (T1) identified 3106 differentially expressed genes common to both stages. GO and KEGG enrichment analyses revealed that these genes were significantly enriched in pathways related to plant hormone signalling, the cytoskeleton, and microtubule organisation. By integrating BSA-Seq and RNA-Seq data, three candidate genes were screened that simultaneously harboured non-synonymous mutations and were significantly highly expressed in the short fruit branch line S14. Combined with bioinformatics analysis, *GH_D02G0744* was predicted to be the most likely key candidate gene regulating cotton fruit branch length. This study provides important genetic resources to elucidate the molecular regulatory mechanisms of cotton fruit branch length and lays a theoretical foundation for molecular breeding to improve cotton plant architecture.

## 1. Introduction

Cotton is a globally significant natural fibre and cash crop, and its yield and quality are directly linked to the development of the textile industry and agricultural economy [1,2]. With the upgrading of the textile industry and the shift toward large-scale, mechanised cotton production, the development of new varieties that are high-yielding, high-quality, and mechanisable has become a key breeding objective [3,4]. Plant architecture is a core trait that influences population photosynthesis, planting density, mechanical operation efficiency, and yield. Among these, fruit branch length, as a key determinant of plant architecture, dictates the spatial distribution of fruit branches and the location of boll formation, playing a decisive role in mechanical harvesting adaptability and yield formation [5,6,7]. Therefore, an in-depth investigation of the key genes that regulate fruit branch length holds significant theoretical and practical value for molecular design breeding and the development of cotton varieties with ideal plant architecture.

Currently, research on identifying genes and molecular mechanisms that govern cotton fruit branch length has made significant progress. In terms of genetic mapping, Wang et al. used a recombinant inbred line (RIL) population to locate a major QTL controlling fruit branch length on chromosome 3, which explained 3.31% of the phenotypic variation. They also found that epistatic QTLs play a crucial role in the genetic regulation of fruit branch length, with their contribution even exceeding that of the major QTL [8]. Chandnani et al. detected eight QTLs associated with fruit branch length in an interspecific backcross population and found that alleles derived from U-cotton significantly influence fruit branch length [9]. Song et al. utilised a BC_1_ population to map five QTLs associated with fruit branch length on chromosome 1 [10]; Zhang used an RIL population to map one QTL for fruit branch length, which explained 3.87–4.26% of the phenotypic variation [11]. Xu et al. used an F_2_ segregating population to map one QTL associated with fruit branch internode length on chromosome A3 [12].

Regarding the elucidation of molecular mechanisms, the central role of hormonal signalling pathways has been confirmed by numerous studies. Li et al. found that gibberellin (GA) and indole-3-acetic acid (IAA) increase fruit branch length by promoting cell elongation, and that abscisic acid (ABA) exhibits a similar effect at specific concentrations [13]; Yang et al. revealed that the *PAG1* gene influences the expression of cell elongation-related genes (such as *GhKCS1*) by regulating endogenous brassinosteroid (BR) levels, thereby controlling fruit branch length [14]; Wu et al. found that the bHLH transcription factor *GhPAS1* positively mediates the BR pathway to affect fruit branch length [15]. Ju et al. confirmed that downregulation of GA synthesis genes inhibits cell elongation, leading to shorter fruit branch internodes [7]; Zhong et al. found that the *GhSBI1* gene inhibits fruit branch elongation by regulating IAA metabolism, reducing active GA levels, and accumulating ABA and jasmonic acid (JA) [16]; Zhan et al. found that the miR164*-GhCUC2-GhBRC1* module activates the *NCED1* gene to promote ABA synthesis and inhibit fruit branch elongation [17]. In addition to hormonal pathways, photoreceptor signalling and epigenetic variations also participate in the regulation of fruit branch length. Li et al. identified the blue light receptor gene *GhFKF1*, which interacts with *GhGI* to influence fruit branch length by regulating the differentiation of axillary meristem tissue [18]; Ji et al. confirmed that copy number variations at the HPDA-D12 locus can upregulate *GhDREB1B* expression and shorten fruit branch length [19]. Overall, existing studies have laid a preliminary theoretical foundation for the molecular genetic research on cotton fruit branch length through genetic mapping, identification of key genes, and analysis of signalling pathways. However, the depth of relevant research remains insufficient, the elucidation of regulatory mechanisms is not systematic enough, and the validation of gene functions is inadequate, which severely limits the precise application of genes related to fruit branch length in molecular breeding for cotton plant architecture.

Previous studies on cotton fruit and branch length have primarily focused on quantitative trait locus (QTL) mapping or transcriptomic analysis. In recent years, the combined application of bulked segregant analysis sequencing (BSA-Seq) and RNA sequencing (RNA-Seq) has emerged as an efficient strategy for rapidly identifying candidate genes for complex crop traits [20]. BSA-Seq enables the rapid identification of genomic regions controlling target traits through whole-genome resequencing of phenotypically extreme bulks [21]; RNA-Seq, in turn, allows for the systematic identification of differentially expressed genes associated with target traits at the transcriptional level [22]. The combination of these two approaches helps further narrow the scope within candidate regions, thereby improving the accuracy and reliability of candidate gene identification. In cotton, Li et al. used BSA-Seq, RNA-Seq, and molecular methods to localise *GhRl4* to chromosome A01 and identified it as a key regulator of round-leaf morphology in the cotton line M113116 [23]. Cui et al. identified the yellow wilt resistance gene *GhDRP* [24]; Wu et al. found that the NAC transcription factor *GbNTL9* influences fibre quality by regulating the arrangement of cellulose microfibrils [25]. Similar strategies have been widely applied to crops such as rice [26,27,28,29,30], maize [31,32,33], and wheat [34,35], yielding significant progress in plant architecture, disease resistance, environmental stress responses, and quality improvement. This fully demonstrates the universality and efficiency of combining BSA-Seq with RNA-Seq in identifying candidate genes for complex quantitative traits.

Currently, research on cotton fruit and branch length based on combined BSA-Seq and RNA-Seq analyses remains limited. Its genetic basis and key regulatory genes have not yet been systematically elucidated, making it difficult to meet the urgent demand for varieties suited to improvements in cotton plant architecture and mechanised production models. The core scientific issue lies in insufficient understanding of the genetic basis and key regulatory genes governing cotton fruit branch length, coupled with a lack of efficient multi-omics-based candidate gene identification strategies, which have hindered progress in molecular breeding for cotton plant architecture suited to mechanised operations. This study hypothesises that differences in cotton fruit branch length are primarily driven by cell elongation, a process regulated by key genes within major QTL regions, and that the combined analysis of BSA-Seq and RNA-Seq can efficiently and accurately identify candidate genes regulating fruit branch length. With the core objectives of elucidating the cytological basis of variations in cotton fruit branch length, mapping QTLs associated with fruit branch length, and screening and preliminarily validating key candidate genes, this study systematically conducted phenotypic characterisation, cytological observations, integrated multi-omics analysis, and preliminary gene expression validation. The aim is to identify genetic resources applicable to marker-assisted selection and molecular design breeding for plant architecture, thereby providing theoretical support for the molecular breeding of cotton with an ideal plant architecture.

## 2. Results

### 2.1. Phenotypic Characteristics of Fruit Branches in Parent and F_2_ Segregation Populations

**Figure 1:**
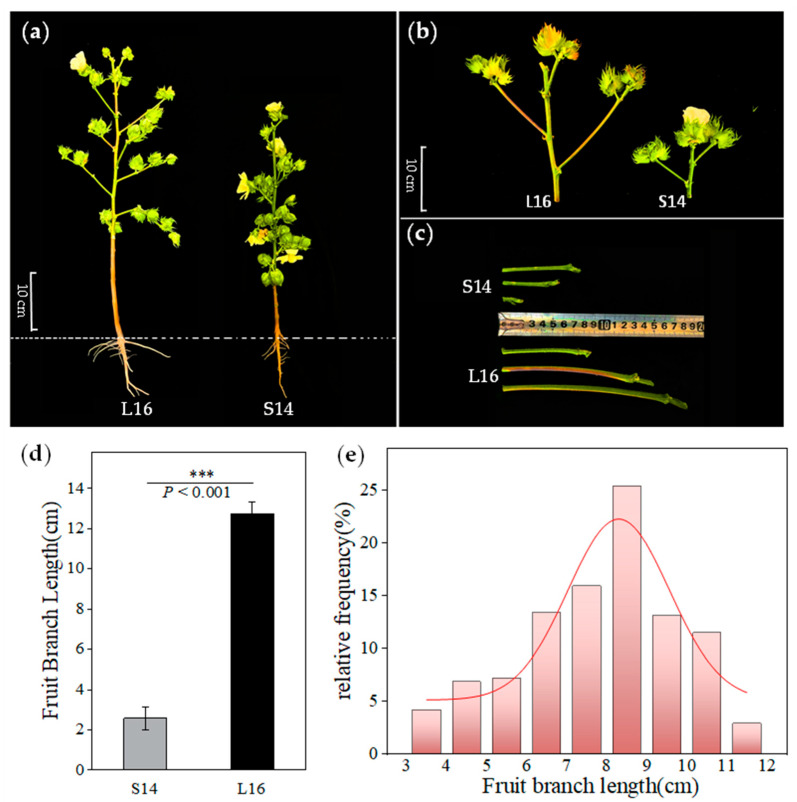
Phenotypic characteristics of parents and the F_2_ population: (**a**) Phenotypic diagram of parental plant architecture. The dotted line indicates the soil surface of the cotton plant; (**b**) Diagram of axillary bud development on parental fruiting branches; (**c**) Phenotypic diagram of parental fruiting branch length; (**d**) Bar chart of parental fruiting branch length; (**e**) Frequency distribution of fruiting branch length in the F_2_ population. Bar graphs represent mean ± standard deviation (n = 30). *** *p* < 0.001.

There was a highly significant difference in fruit branch length between the parental lines L16 and S14 (Figure 1a–c). Measurements and analysis of the fifth fruit branch length in both parental lines showed that the fruit branches of the long-branch line L16 were significantly longer than those of the short-branch line S14. The average fruit branch length for L16 was 12.75 cm, while that for S14 was 2.57 cm; the fruit branch length of L16 was 4.96 times as long as that of S14 (Figure 1d). Analysis of the frequency distribution of fruit branch length in the F_2_ population revealed that this trait exhibits a continuous distribution, consistent with the genetic characteristics of a quantitative trait (Figure 1e).

### 2.2. Dynamics of Fruit Branch Internode Elongation

**Figure 2:**
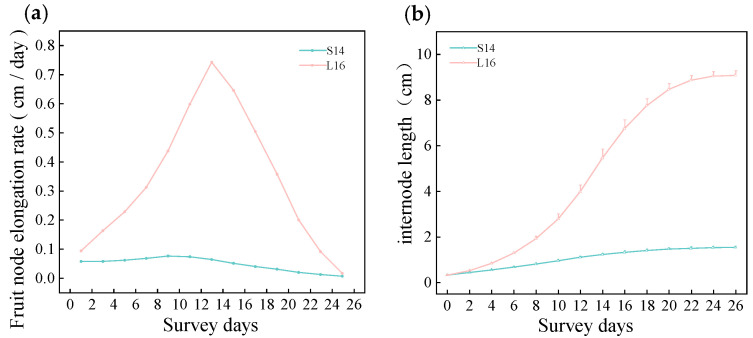
Dynamics of internode elongation in parental fruit branches: (**a**) Internode elongation rate curve; (**b**) Internode length versus time curve.

The internode elongation rate of L16 fruit branches exhibited a single-peak curve with an initial rise followed by a decline over time, reaching a maximum elongation rate of 0.74 cm·d^−1^ on day 13. In contrast, the elongation rate of S14 fruit branches remained consistently low with a gradual change, peaking at a mere 0.08 cm·d^−1^ on day 9 (Figure 2a). In terms of length dynamics, the internode elongation of L16 fruit branches exhibited an S-shaped curve, reaching 9.08 cm by day 28, whereas S14 branches only reached 1.52 cm, resulting in a final length difference of 5.97-fold (Figure 2b). These findings indicate that differences in fruit branch internode length primarily stem from variations in elongation rates.

### 2.3. Paraffin Section and Cytological Analysis

**Figure 3:**
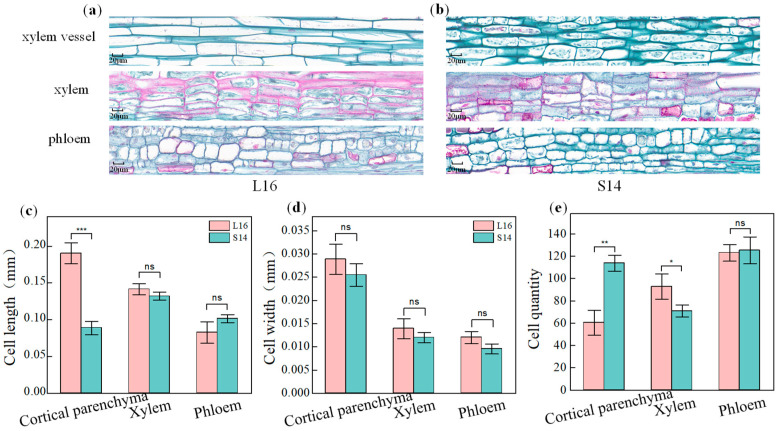
Comparison of cytological characteristics in longitudinal sections of L16 and S14 fruit internodes. (**a**,**b**) Paraffin sections of cortical parenchyma cells, xylem cells, and phloem cells in L16 and S14; Scale bar = 20 μm. (**c**) Cell length statistics for L16 and S14; (**d**) Cell width statistics for L16 and S14; (**e**) Cell count per unit length (10 mm) for L16 and S14. Bar graphs represent mean ± standard deviation (n = 5); * *p* < 0.05, ** *p* < 0.01, *** *p* < 0.001, ns, not significant (*p* ≥ 0.05).

To investigate the cytological basis for differences in fruit branch length, longitudinal paraffin sections of the internodes of the long fruit branch L16 and the short fruit branch S14 were examined and analysed. The results (Figure 3c,d) showed that the length of cortical parenchyma cells in L16 was significantly greater than in S14 (*p* < 0.001), with the difference exceeding twofold. In contrast, there were no significant differences in cell length between the two materials in the xylem and phloem (*p* > 0.05). Furthermore, there were no significant differences in the width of cortical parenchyma cells, xylem cells, or phloem cells between L16 and S14 (*p* > 0.05), indicating that radial cell growth is not the cause of the differences in fruit branch length. Statistical analysis of cell numbers per unit length (Figure 3e) showed that the number of cortical parenchyma cells in the short fruit branch S14 was extremely significantly higher than that in the long fruit branch L16 (*p* < 0.01). In comparison, the number of xylem cells in L16 was significantly higher than that in S14 (*p* < 0.05). There was no significant difference in the number of phloem cells between the two, and the morphological and arrangement characteristics of the cells in the sections were fully consistent with the quantitative results (Figure 3a,b). The results indicate that the longitudinal elongation of cortical parenchyma cells, rather than cell division, is the primary cytological factor underlying the differences in internode length between cotton fruit branches.

### 2.4. BSA-Seq Analysis and Candidate Region Localisation

To identify genetic loci regulating fruit-bearing shoot length, BSA-seq resequencing was performed on L16, S14, and F_2_ extreme pools LB and SB. A total of 321.84 GB of raw data was obtained, with Q20 ≥ 99.09% and Q30 ≥ 96.76%, and GC content ranging from 35.29% to 35.58% (Table S1), indicating high-quality sequencing. Following quality control, 312.48 GB of clean data were obtained (Table S2). Alignment of the clean data against the reference genome yielded 2,091,128,641 alignments, with a sample alignment rate of 99.68–99.71%. The 1× and 4× genome coverage percentages were 99.40% and 98.58%, respectively, indicating good uniformity of coverage. The average sequencing depth for parental lines was 24×, while offspring averaged approximately 32× (Table 1).

**Figure 4:**
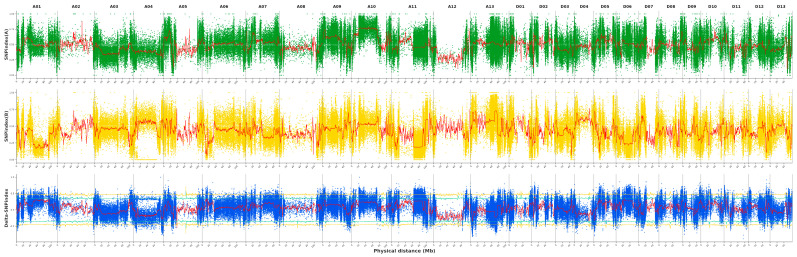
SNP-index mapping of cotton fruit branch length. The x-axis denotes chromosome names and lengths, the y-axis represents SNP-index values, and the red line indicates the mean SNP-index within the window. In contrast, the orange and green lines denote the 99% and 95% confidence intervals, respectively. Chromosomal distribution of Delta_SNP_index correlation values.

Following variant detection and annotation, a total of 3,598,394 single-nucleotide polymorphisms (SNPs) and 561,519 insertions and deletions (InDels) were identified (Table S3). Association analysis was performed using the SNP-index algorithm, and Δ(SNP-index) values were calculated (Figure 4) and screened based on 95% confidence intervals. Ultimately, SNPs were identified on chromosomes A01 (21,200,001–23,800,000 bp), A06 (12,800,001–14,800,000 bp), D02 (6,800,001–12,400,000 bp), D07 (1–1,000,000 bp), and D11 (1–2,800,000 bp), with a total of 82 coding genes annotated within these regions.

### 2.5. RNA-Seq Analysis and Identification of Differentially Expressed Genes

**Figure 5:**
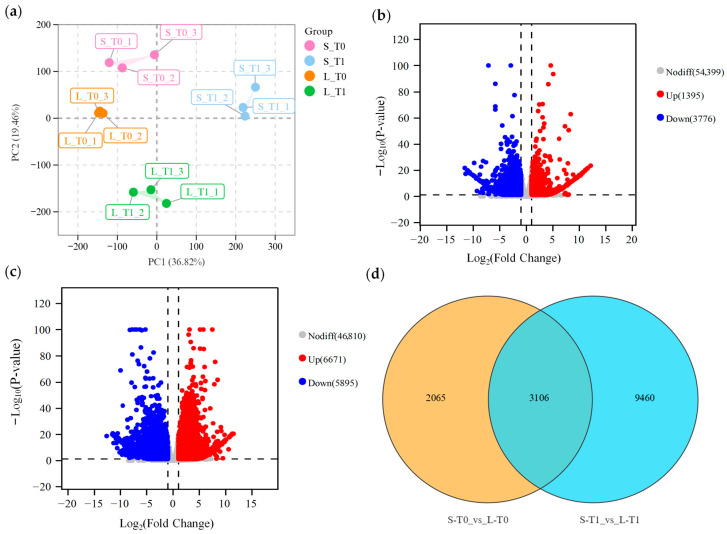
Differentially expressed gene analysis between L16 and S14 at T0 and T1 time points: (**a**) Principal component analysis (PCA) of sample expression levels; (**b**) Volcano plot of differentially expressed genes between L16 and S14 at T0; (**c**) Volcano plot of differentially expressed genes between L16 and S14 at T1; (**d**) Venn diagram of differentially expressed genes common to and specific to both T0 and T1 time points.

To investigate expression differences between long and short fruit-bearing branches during development, RNA-Seq analysis was performed on internode tissue from the first fruit node of the fifth fruit-bearing branch at T0 and T1 stages for L16 and S14. Each library generated 37–58 million raw reads. The proportion of high-quality bases exceeded 95.75% across all samples, with an average GC content of approximately 43.21% (Table S4). Following quality control, the mapping rate of clean reads to the reference genome exceeded 96.29% (Table S5). Principal component analysis (PCA) revealed that biological replicates from the same material and developmental stage clustered closely, whilst replicates from different materials or developmental stages separated clearly (Figure 5a), indicating stable and reliable RNA-Seq data.

The number of differentially expressed genes associated with fruit-bearing shoot length between L16 and S14 during the T0 and T1 periods exhibited significant differences. During the T0 period, a total of 5170 differentially expressed genes were identified, comprising 1395 upregulated and 3776 downregulated genes (Figure 5b). At T1, 12,566 differentially expressed genes were identified, comprising 6671 upregulated and 5895 downregulated genes (Figure 5c). Venn analysis revealed 3106 genes differentially expressed between the two periods (Figure 5d).

**Figure 6:**
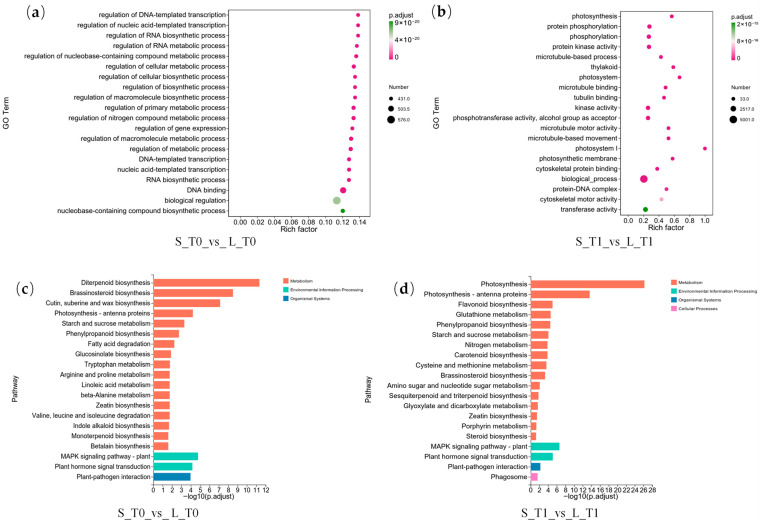
GO and KEGG enrichment analysis of differentially expressed genes. (**a**) GO enrichment for S_T0_vs_L_T0 group; (**b**) GO enrichment for S_T1_vs_L_T1 group; (**c**) KEGG enrichment for S_T0_vs_L_T0 group; (**d**) KEGG enrichment for S_T1_vs_L_T1 group.

GO enrichment analysis of differentially expressed genes during T0 and T1 periods revealed (Figure 6a,b) that T0-period genes were predominantly enriched in fundamental biological processes such as transcriptional regulation, primary metabolism, and biosynthesis. In contrast, T1-stage differentially expressed genes showed significant enrichment in photosynthesis, energy metabolism, protein phosphorylation, microtubule organisation, and cytoskeletal processes. This reflects the fruit-bearing branch’s demands for energy supply, signal transduction, and cellular structural remodelling during rapid elongation. KEGG enrichment analysis results (Figure 6c,d) showed that differentially expressed genes from both stages were significantly enriched in pathways including plant hormone signal transduction, MAPK signalling, phenylpropanoid metabolism, and starch and sucrose metabolism. This indicates that hormone signalling and carbon metabolism play crucial roles in fruit branch development.

### 2.6. Combined Analysis of BSA-Seq and RNA-Seq for Screening Candidate Genes

**Figure 7:**
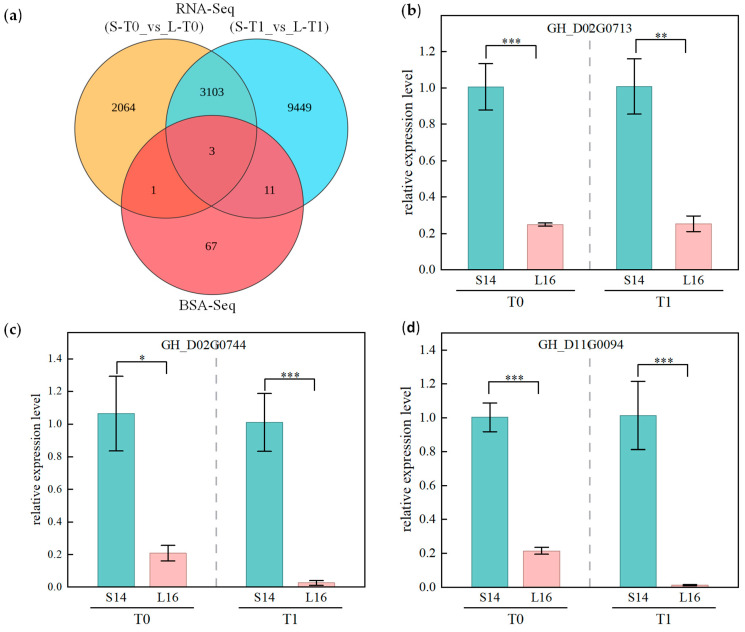
Candidate genes identified by combined BSA-Seq and RNA-Seq analysis and qRT-PCR validation results. (**a**) Venn diagram of candidate genes; (**b**–**d**) qRT-PCR expression analysis of three candidate genes in L16 and S14. Note: The x-axis represents the sample (L16, S14), and the y-axis represents relative expression levels (calculated using the 2^−ΔΔCt^ method); * *p* < 0.05, ** *p* < 0.01, *** *p* < 0.001.

To further narrow down the range of key candidate genes within the QTL intervals identified by BSA-Seq, this study integrated genetic mapping results with transcriptomic data for a joint analysis. We performed intersection analyses of the 82 coding genes within the BSA-Seq-mapped intervals with the significantly differentially expressed genes in parental lines L16 and S14 at T0 and T1. The results showed that a total of 4 genes within the QTL interval exhibited significant inter-parental expression differences at the T0 stage, and 14 genes exhibited significant inter-parental expression differences at the T1 stage; among these, only 3 genes demonstrated stable, significant inter-parental expression differences at both the T0 and T1 stages, representing the common intersection of coding genes within the QTL interval and differentially expressed genes across the two stages. Further analysis of sequence variations between parental lines for these three candidate genes revealed that all three genes harboured non-synonymous mutation sites between L16 and S14, specifically *GH_D02G0713*, *GH_D02G0744*, and *GH_D11G0094* (Figure 7a, Table 2 and Table S6).

Functional annotation results show that *GH_D02G0713* encodes an extensin-like protein; *GH_D02G0744* encodes the LRR receptor-like serine/threonine protein kinase GSO1; and *GH_D11G0094* encodes a protein DETOXIFICATION 49-like. RNA-Seq analysis indicates that these three genes were significantly highly expressed in the short fruit branch material S14 during both the T0 and T1 periods. Specifically, the average expression levels of *GH_D02G0713* in S14 during the T0 and T1 periods were 2.47-fold and 4.18-fold higher than those in L16, respectively; the average expression levels of *GH_D02G0744* in S14 during the T0 and T1 periods were 5.71-fold and 15.53-fold higher than those in L16, respectively; For *GH_D11G0094*, the average expression levels in S14 and L16 at the T0 time point were 0.37 and 0.13, respectively, while the average expression level in S14 at the T1 time point rose to 1.68; however, no expression was detected in any of the three biological replicates in L16.

### 2.7. qRT-PCR Validation of Differentially Expressed Genes

To validate the reliability of the transcriptomic analysis and further elucidate the expression characteristics of the candidate genes, qRT-PCR was performed on the three candidate genes. Results demonstrated (Figure 7b–d) that the expression levels of all three candidate genes were significantly higher in the short-fruit-branch material S14 compared to the long-fruit-branch material L16 (*p* < 0.001). Furthermore, their expression trends aligned with RNA-Seq data, providing high confidence in the transcriptome and a reliable foundation for subsequent functional validation and mechanistic studies.

## 3. Discussion

### 3.1. Biological and Cytological Basis of Fruit Branch Length Variation

In this study, fruit branch length in the F_2_ population exhibited a continuous distribution, consistent with the genetic patterns of typical quantitative traits, indicating that fruit branch length is controlled by the synergistic action of multiple genes. This finding is consistent with previous research on the genetic characteristics of fruit branch internode length in cotton [36]. Further analysis of the developmental dynamics of fruit branch internodes revealed that the length difference between long- and short-fruit branch materials primarily stems from differences in fruit branch elongation rates, which is generally consistent with existing research conclusions on the dynamic changes in fruit branch internode elongation [16,37]. At the cytological level, paraffin section results provided direct evidence of differences in fruit branch length. Cytological observations and analyses of the internodes of the long fruit branch accession L16 and the short fruit branch accession S14 clarified the core cytological mechanisms underlying the differences in fruit branch length. The study found that the elongation of cotton fruit branch internodes is not primarily driven by radial expansion of cells or the morphogenesis of vascular tissue cells; nor can the increase in cell number resulting from cell division effectively drive significant internode elongation. The longitudinal elongation capacity of cortical parenchyma cells is the core regulatory factor determining fruit branch internode length. The extremely strong longitudinal elongation capacity of cortical parenchyma cells in the long fruit branch material directly drove internode elongation. In contrast, in the short fruit branch material, the longitudinal elongation of cortical parenchyma cells was significantly inhibited. Even though increased cell division increased cell number, it could not compensate for the restriction of internode growth due to insufficient cell elongation, ultimately resulting in the short fruit branch phenotype. These results are consistent with the classical developmental theory that plant organ growth is regulated by the synergistic interaction of cell division and cell expansion, and align with the widely accepted view that cell elongation is the key factor determining the length of plant stems and fruit branch internodes [7,14,16,37], providing direct cytological evidence for the formation of cotton fruit branch architecture. It is worth noting that previous studies have indicated that xylem cell length is a key factor regulating fruit branch length [16]. However, in this study, the most significant cytological differences between long and short fruit branches were observed in the cortical parenchyma cells. At the same time, there were no significant differences in the development of vascular tissue cells. This suggests that the dominant cell types regulating fruit branch internode elongation vary among cotton materials with different genetic backgrounds, and that the cytological regulatory mechanisms governing cotton fruit branch length exhibit significant specificity and diversity. This study elucidated differences between long- and short-fruiting materials in terms of cortical parenchyma cell elongation, offering a new avenue for linking cytological characteristics to molecular regulatory networks.

### 3.2. QTL Interval Mapping and Characteristics of Differentially Expressed Genes

The BSA-Seq technique enables rapid, genome-wide identification of candidate regions associated with target traits by performing whole-genome resequencing of mixed plant pools with extreme phenotypes. This technique has been successfully applied to the genetic analysis of important traits in various crops, including pumpkin fruit length, rice plant height, and oyster mushroom stem length [38,39,40]. Previous studies have identified multiple QTLs associated with fruit branch length in cotton, located on chromosomes A04, A11, D03, D07 [10], A03 [12], and D01 [16], further highlighting the high complexity of this trait. In this study, using BSA-Seq technology, we conducted QTL mapping for fruit branch length in an F_2_ segregating population derived from the long-fruit-branch material L16 and the short-fruit-branch material S14 of upland cotton. A total of five candidate regions were identified on chromosomes A01, A06, D02, D07, and D11, with a combined length of approximately 14 Mb, containing 82 annotated genes. These genome-wide results confirm that multiple genes control cotton fruit branch length and further enrich the genetic basis of this trait, building upon existing QTL maps.

In terms of chromosomal distribution, the significant signal on chromosome D07 partially overlaps with previously reported QTLs associated with fruit branch length [10], which provides corroborative evidence for the reliability of the localisation results in this study. In contrast, the candidate regions on chromosomes A01, A06, D02, and D11 have rarely been reported in the existing literature, demonstrating the innovative nature of this study in expanding the understanding of regulatory loci for cotton fruit branch length. Among the five candidate regions, the region on chromosome D02 spans 5.6 Mb, making it the longest region with the highest number of non-synonymous mutant genes. It is the core candidate region for regulating cotton fruit branch length and is also the primary reason the two candidate genes identified in subsequent joint analyses were located on this chromosome. In future studies, we will develop KASP molecular markers for the key QTL regions on chromosome D02 and construct a larger F_2:3_ population to narrow the candidate regions through fine mapping further. The four fruit-branch-length-related QTLs newly mapped in this study enrich the genetic loci for cotton fruit-branch length and provide new targets for marker-assisted breeding. BSA-Seq analysis not only validated some known plant architecture regulatory loci but also identified multiple new candidate regions, providing potential genetic resources for the molecular improvement of cotton fruit branch length. In subsequent studies, we will develop KASP molecular markers for the key QTL regions on chromosome D02 and construct a larger F_2:3_ population for fine mapping to further narrow the candidate regions.

Traditional QTL mapping often struggles to rapidly identify key genes due to large genetic intervals and numerous candidate genes. Combining BSA-Seq with RNA-Seq enables rapid screening of functionally relevant genes within a specific mapping interval based on expression differences. It has become an important technical approach for elucidating complex quantitative traits. This study systematically compared transcriptomic differences between the long-shoot material L16 and the short-shoot material S14 during the shoot initiation stage (T0) and the rapid elongation stage (T1). A total of 5170 and 12,566 differentially expressed genes (DEGs) were identified during the T0 and T1 periods, respectively. The number of DEGs in T1 was approximately 2.4 times that of T0, indicating that the rapid elongation phase is a critical period for the significant phenotypic differences in fruit branches. This finding is highly consistent with the conclusion from phenotypic analysis that “the difference in fruit branch length between the two parents primarily stems from differences in elongation rates during the rapid elongation phase.” Compared with existing cotton fruit branch transcriptomic studies, this study simultaneously covered both the initiation and rapid elongation phases. The 3106 commonly differentially expressed genes identified in this study eliminate developmental-stage-specific confounders and more accurately reflect the core regulatory genes stably associated with fruit branch length. Due to experimental design constraints, RNA-Seq sampling in this study was limited to two key developmental stages. In future studies, we will increase the number of sampling time points to systematically elucidate the dynamic transcriptional regulatory networks during fruit branch elongation.

KEGG enrichment analysis revealed that differentially expressed genes between the two periods were significantly enriched in plant hormone signalling pathways, which is consistent with previously reported findings that GA, IAA, and BR hormones regulate cotton fruit branch elongation. Numerous studies have confirmed that multiple plant hormones synergistically regulate the length of cotton fruit branches. Among these, IAA, GA, cytokinins (CTK), and BR generally increase fruit branch length by promoting cell division and elongation, whereas ABA, ethylene (ET), and JA typically exert inhibitory effects. These hormones participate in the formation of plant architecture by influencing cell wall softening, the cytoskeleton, and the activity of related transcription factors [7,14,15,16,17,41]. It is worth emphasising that the number of differentially expressed genes at the T1 stage was significantly higher than at the T0 stage, and that these genes were primarily associated with microtubule organisation and cell wall synthesis-related pathways. This further confirms, at the transcriptomic level, that the longitudinal elongation of cortical parenchyma cells constitutes the cytological basis for differences in fruit branch length. Combined with the differences in cortical parenchyma cell length observed in paraffin sections, it can be inferred that differences in the activity of hormonal signalling pathways and their downstream regulation of the cell wall and cytoskeleton among different materials may be key factors leading to variations in the extent of longitudinal elongation of cortical parenchyma cells, which in turn result in differences in fruit branch length. This inference is highly consistent with existing research on the role of hormones in regulating cotton plant architecture. Further, it confirms, at the transcriptomic level, the central role of plant hormone signalling networks in the formation of fruit branch length. The transcriptome analysis identified numerous differentially expressed genes associated with fruit-to-branch length. Still, it was not possible to determine whether these genes were located within the QTL regions identified by BSA-Seq. A combined omics analysis can identify key candidate genes at both the sequence-variation and expression-regulation levels, thereby significantly improving the accuracy of the screening process.

### 3.3. Functional Inference and Validation of Candidate Genes for Fruit Branch Length

The genetic analysis of complex agronomic traits relies on efficient gene-mapping and functional validation strategies [42]. The combined application of BSA-Seq and RNA-Seq has been extensively used in rice, maize, wheat, and other crops to identify genes associated with agronomic traits, stress resistance, and quality characteristics [26,27,28,29,30,31,32,33,34,35]. In this study, BSA-Seq was used to narrow the candidate pool from the entire genome to 82 genes. Subsequently, differential expression analysis via RNA-Seq ultimately identified three candidate genes: *GH_D02G0713*, *GH_D02G0744*, and *GH_D11G0094*. qRT-PCR validation revealed that these genes were significantly highly expressed in the short-branching line S14. In contrast, their expression levels were lower in the long-branching line L16, and they exhibited stable differences in expression across two developmental stages. Given that S14 exhibits a short fruit branch phenotype, this pattern is more consistent with the characteristics of a negative regulator; that is, high expression of these genes may inhibit the longitudinal elongation of cortical parenchyma cells, thereby leading to shorter cotton fruit branches. This is consistent with the previous cytological observation that the longitudinal length of cortical parenchyma cells in L16 was significantly longer than in S14, providing histological evidence supporting the gene function hypothesis.

Among these, *GH_D02G0713* encodes an extensin-like protein, a member of the family of hydroxyproline-rich cell-wall glycoproteins in higher plants [43]. These proteins are extensively involved in cell elongation, cell wall construction, and developmental regulation [44,45]. Multiple studies indicate that elevated expression levels of certain extensin family members correlate with inhibited plant cell elongation. For instance, overexpression of BnPERK1 in Arabidopsis resulted in weakened apical dominance and increased branching [46]; overexpression of AtEXT1 led to a significant reduction in plant height [47]; and overexpression of OsEXTL in rice inhibited internode cell elongation, causing reduced plant height [43]. These results collectively indicate that members of the extensin family play a crucial regulatory role in organ elongation by altering the mechanical properties of the cell wall. In this study, *GH_D02G0713* was significantly upregulated in the short fruit branch material S14. Its high expression may lead to excessive cross-linking of the cell wall, increased rigidity, and reduced extensibility, thereby inhibiting the longitudinal elongation of cortical parenchyma cells and negatively regulating fruit branch length. This hypothesis is highly consistent with the histological observation that “cortical parenchyma cells are shorter” in the short fruit branch material, as revealed by paraffin sections.

*GH_D02G0744* encodes the LRR receptor-like serine/threonine protein kinase GSO1, which belongs to the LRR-RLK family [48]. Members of this family are extensively involved in perceiving extracellular signals and interact closely with hormone signalling pathways, such as IAA and BR, playing conserved yet diverse roles in the regulation of plant architecture and organ development. The tomato short-internode and short-pedicel gene encodes an LRR receptor-like serine/threonine protein kinase, ERECT, which regulates stem elongation by modulating GA metabolism [49]; a loss-of-function mutation in the cucumber CsERECTA gene leads to reduced cell numbers and a compact plant architecture [50]; members of the maize ZmERECTA family exhibit functional redundancy, with ZmER1 playing a dominant role; its mutants display multiple phenotypes, including a compact plant architecture [51]; and rice LRK1 inhibits internode elongation by downregulating the expression of genes involved in GA biosynthesis [52]. These studies indicate that the LRR-RLK family plays a crucial role in internode elongation and plant architecture in crops. In this study, the expression level of *GH_D02G0744* in S14 was significantly higher than that in L16 at both the T0 and T1 time points. Combined with the functional background of its receptor kinase and the significant enrichment of hormone-signal-related DEGs, it is hypothesised that *GH_D02G0744* may act as a receptor or co-receptor for hormone (e.g., BR or GA) signalling. By modulating cellular sensitivity to hormonal signals or the intensity of signal transduction, it influences the expression of downstream genes related to cell wall remodelling, thereby negatively regulating the elongation of cortical parenchyma cells and resulting in shorter fruit branches. Compared to *GH_D02G0713*, *GH_D02G0744* exhibits a greater fold change in expression and is likely associated with hormone signalling pathways. Therefore, it is the most promising candidate gene for subsequent functional validation.

*GH_D11G0094* encodes a DETOXIFICATION 49-like protein. Existing studies have largely associated this class of proteins with pathogen stress and environmental responses [53]. However, clear evidence of their direct role in regulating plant growth and development is still lacking. In this study, the overall expression levels of *GH_D11G0094* were low in S14 and L16, with only a slight increase observed during the T1 stage of S14. Therefore, its role in regulating fruit branch length requires further experimental validation, such as through gene overexpression, RNAi, or CRISPR/Cas9, to clarify its function.

By integrating QTL mapping data, gene expression pattern analysis, and functional annotation information, this study identified *GH_D02G0713* and *GH_D02G0744* as key candidate genes regulating cotton fruit branch length. Among these, *GH_D02G0744* exhibits significant expression differences at the same developmental stage across materials with varying fruit branch lengths. It possesses a clear functional background within the receptor kinase family, making it a priority candidate for subsequent functional validation. This study still has certain limitations. Functional validation experiments, such as VIGS and QTL interval fine-mapping, have not yet been conducted. Furthermore, functional inference for the candidate genes is primarily based on homologous gene annotation, and systematic validation of their functions and genetic effects is lacking. Subsequent studies will focus on systematic investigations of target loci and candidate genes. We will develop KASP markers for the target QTL regions and perform fine-mapping using expanded populations. By combining allelic association analyses in natural populations, we will clarify the genetic effects of candidate genes. Additionally, we will utilise VIGS, CRISPR/Cas9 gene editing, and transgenic overexpression technologies to systematically elucidate the biological functions and molecular mechanisms by which these candidate genes regulate cotton fruit branch length and to clarify their interactions with hormone signalling and cell wall remodelling during fruit branch development. The candidate genes and their superior allelic variants identified in this study can be integrated into a molecular design breeding system for cotton plant architecture. This will provide important genetic resources and theoretical support for the targeted improvement of fruit branch length and the development of cotton varieties with ideal plant architecture suited for high-density planting and mechanised operations.

## 4. Materials and Methods

### 4.1. Experimental Materials and Population Construction

In this study, a genetic segregation population was established using the upland cotton long-fruit-branch line 2203-16 (L16)—which was independently bred and continuously inbred over multiple generations in the Xinjiang cotton-growing region—as the female parent, and the short-fruit-branch line 9293-14 (S14) as the male parent. Multi-year, multi-location field phenotypic evaluations revealed that the average boll length of L16 was consistently 12–14 cm, while that of S14 was consistently 2–4 cm, with the difference between the two reaching a highly Figure 4 significant level (*p* < 0.001); furthermore, key agronomic traits such as growth period, yield components, and fiber quality were consistent between the two, minimizing genetic background interference from non-target traits, making them excellent parental lines for elucidating the genetic regulatory mechanisms of cotton fruit branch length. The F_1_ generation was obtained from crosses in 2021; the F_2_ segregating population was derived from inbred F_1_ crosses in 2022; and in 2023, an F_2_ population comprising 320 individual plants was planted, and phenotypic characterisation and genetic analysis were completed. All materials were grown at the Kuche Upland Cotton Experimental Base of the Xinjiang Academy of Agricultural Sciences (41°40′ N, 82°56′ E), with field management following local standard cultivation protocols. The parental lines possess homozygous, stable genetic backgrounds and exhibit extreme differences in the target trait, providing a reliable genetic foundation for subsequent QTL mapping and the identification of key genes associated with cotton fruit-branch length.

### 4.2. Fruit Branch Internode Elongation and Fruit Branch Length Measurement

During bud break, individual plants of L16 and S14 with consistent growth were selected. Based on multi-year phenotypic assessment results, the length of the fifth fruiting branch was determined to adequately represent the average length of fruiting branches across the entire plant. Using the new growth point of the fifth fruiting branch as the marker, the length of the first fruiting node was measured every two days using a vernier calliper (accuracy 0.1 mm) until elongation essentially ceased. Growth rate curves and dynamic elongation patterns were plotted to compare differences between the two parents in fruiting branch elongation rates and patterns. At maturity, the fruiting branch lengths of the F_2_ population were surveyed to provide phenotypic data for subsequent genetic analysis.

### 4.3. Paraffin Sections and Histological Examination

During the rapid elongation phase of the fifth fruit-bearing shoot of parental lines L16 and S14, tissue samples were collected from the first internode of the fifth fruit-bearing shoot. Immediately after sampling, the specimens were placed in FAA fixative (70% ethanol: 38% formalin: glacial acetic acid = 18:1:1) for 24 h; after fixation, the samples were sequentially dehydrated using a gradient ethanol solution, paraffin-embedded, and, after cooling the paraffin blocks to −20 °C, sectioned into 4-μm-thick sections. After dewaxing to water, the sections were stained with eosin for 2 min, decolourised with a gradient ethanol solution, and counterstained with hematoxylin for 15 s. Following staining, the sections were dehydrated with anhydrous ethanol, cleared with xylene, and mounted with neutral resin. The sections were scanned with an LG-S80 scanner (Servicebio, Wuhan, China), and high-resolution images of the cortex, xylem, and phloem regions were selected for observation and analysis using Saiviewer software (version 1.0.9). Image-Pro Plus 6.0 was used to measure cell length and width in millimetres in the target areas. The number of cells within a 10-mm length range was counted, and the average was calculated. This experiment included 3 biological replicates, each with 5 technical replicates. Data are presented as mean ± standard deviation. An independent-samples *t*-test was used to analyze differences between groups, with a significance level of *p* < 0.05.

### 4.4. Pool Construction and Whole-Genome Resequencing

From the F_2_ population, 30 plants exhibiting extremely long fruit-bearing branches and 30 plants exhibiting extremely short fruit-bearing branches were selected. Concurrently, tender leaves were collected from parental lines L16 and S14 as test materials, rapidly frozen in liquid nitrogen, and stored at −80 °C. Genomic DNA was extracted using a modified CTAB method. Following quality control, equal amounts of DNA from extreme long-fruit and short-fruit branches were pooled to construct the long-fruit branch pool (LB) and short-fruit branch pool (SB), alongside parent pools for L16 and S14. Genomic DNA was randomly sheared to 350 bp fragments via ultrasonication, followed by end repair. An A base was added to the 3′ end of each fragment, and an Index-bearing sequencing adapter was ligated to the 5′ end. After removing adapter dimers and purifying the library, PCR amplification, fragment selection, and purification yielded a sequencing library with approximately 450 bp inserts. Following quality control, the library underwent paired-end sequencing (PE150) on the Illumina NovaSeq™ X Plus platform. Target sequencing depth was approximately 20× for the parental pool and 30× for the hybrid pool.

The raw data obtained from sequencing undergo quality assessment and filtering to remove adapter contamination, low-quality sequences, and sequences with a high proportion of ambiguous bases, resulting in high-quality, clean data. The Clean Data was aligned to the reference genome of the upland cotton standard line TM-1 (https://www.cottongen.org/species/Gossypium_hirsutum/ZJU-AD1_v2.1 (accessed on 12 August 2025)) using the BWA software [35]. The Picard software (version 2.23.9) [54] was used to sort the aligned sequences by genomic coordinates and remove PCR-induced duplicates. Using the GATK software (version 3.8) [55] to perform local re-alignment of InDel regions and genome-wide SNP/InDel variant detection, and the ANNOVAR software (https://annovar.openbioinformatics.org/) [56] was used for functional annotation of variant sites. Variant sites were rigorously filtered to remove those with sequencing depths below 5× in both parental and offspring pools, retaining only valid sites where parental genotypes were homozygous and stable polymorphic differences were present. Association analysis was performed using the SNP-index (InDel-index) algorithm, with a 2 Mb sliding window and a 200 kb step size. The average SNP-index for each window in the LB and SB pools was calculated separately, and the difference between the SNP-index values of SB and LB was used to obtain Δ(SNP-index). After 10,000 permutations, genomic regions significantly associated with differences in fruit branch length were identified using a 95% confidence level as the threshold.

### 4.5. RNA-Seq Sequencing Analysis

At the onset of fruit branch formation (T0, when fruit branch primordia first become visible) and the rapid elongation phase (T1, approximately day 13 for L16 and day 9 for S14), tissue samples were collected from the first internode of the fifth fruit branch of L16 and S14, respectively. The samples were flash-frozen in liquid nitrogen and stored at −80 °C, with three biological replicates per sample and per time point. Total RNA was extracted using the DP762-T1A Magnetic Tissue Total RNA Kit (Tiangen Biochemical Technology, Beijing, China) after quality verification using a NanoDrop spectrophotometer (Thermo Scientific, Waltham, MA, USA) and an Agilent 2100 Bioanalyzer (Agilent Technologies Inc., Culver City, CA, USA). RNA-Seq libraries were then constructed and sequenced on the Illumina NovaSeq™ X Plus platform. Raw sequencing data were processed using fastp [57] and FastQC [58] for quality control and filtering to obtain high-quality, clean data. HISAT2 [59] was used to align the clean reads to the upland cotton reference genome TM-1 V 2.1, and HTSeq [60] was used to quantify gene expression and calculate FPKM values. Based on the expression quantification results, DESeq2 [61] was used to identify differentially expressed genes, with the screening criteria set at *p* ≤ 0.05 and |log_2_FoldChange| > 1. Finally, clusterProfiler [62] was used to perform GO functional annotation and KEGG pathway enrichment analysis on the differentially expressed genes. All enrichment analyses were corrected for multiple comparisons using the Benjamini–Hochberg (BH) method, with a post-correction *p*-value threshold set at 0.05. The visualisation results display the top 20 entries with the most significant post-correction *p*-values in each category.

### 4.6. Real-Time Quantitative Reverse Transcription Polymerase Chain Reaction (qRT-PCR) Validation

The three differentially expressed genes identified by joint analysis were validated by qRT-PCR, using *GhActin14* as the housekeeping gene (GenBank accession number: AY305733) [16]. Total RNA meeting quality control standards from the RNA-Seq analysis was selected. cDNA was synthesised via reverse transcription using the Advantage^®^ RT-for-PCR Kit (Clontech, Mountain View, CA, USA). Primers were designed using Primer3, and PCR reactions were performed with AceQ^®^ qPCR SYBR^®^ Green Master Mix (Vazyme Biotech Co., Ltd., Nanjing, China), with amplification performed on a real-time fluorescent quantitative PCR instrument. Primer specificity was assessed via amplification and melting curve analysis, and relative expression levels were calculated using the 2^^(−ΔΔCt)^ method. Each gene was amplified in triplicate biological replicates, with three technical replicates per replicate. Primer sequences are provided in Table S7.

## 5. Conclusions

Cotton fruit branch length is a key trait that determines plant architecture and suitability for mechanised operations; identifying relevant candidate genes is crucial for breeding varieties with ideal plant architecture suited for mechanised production. This study demonstrated through histological analysis that the longitudinal elongation of cortical parenchyma cells primarily determines differences in fruit branch length. Using an F_2_ segregating population and BSA-Seq technology, five QTLs associated with fruit branch length were mapped, and the regions contained 82 coding genes. Further integration of RNA-Seq and qRT-PCR validation identified three candidate genes associated with fruit branch length. Among these, the receptor kinase gene *GH_D02G0744* was identified as the most promising candidate gene for fruit branch length due to its significant expression differences between long and short materials and its well-defined functional background. Subsequent studies will elucidate its regulatory mechanisms through methods such as transgenic analysis. These findings provide cytological evidence for elucidating cotton fruit branch elongation and offer important genetic resources and theoretical support for improving plant architecture and breeding varieties suitable for mechanised operations.

## Figures and Tables

**Table 1:** BSA reference genome alignment data statistics.

Sample	Total Reads	Mapped Reads	Mapping Rate (%)	Average Depth (×)	Coverage 1× (%)	Coverage 4× (%)
P14	426,935,534	425,684,860	99.71	23.16	99.18	98.18
P16	457,298,273	455,862,218	99.69	24.62	99.26	98.32
LB	543,182,805	541,581,351	99.71	28.28	99.54	98.76
SB	670,145,412	668,000,212	99.68	35.31	99.65	99.06

**Table 2:** Candidate genes associated with fruit branch length identified through combined BSA-Seq and RNA-Seq analysis.

Gene_ID	Homologous Gene in Arabidopsis	Functional Annotation
*GH_D02G0713*	*AT1G21310*	extensin-like
*GH_D02G0744*	*AT2G34930*	LRR receptor-like serine/threonine-protein kinase GSO1
*GH_D11G0094*	*AT4G23030*	protein DETOXIFICATION 49-like

## Data Availability

The original contributions presented in this study are included in the article/Supplementary Material. Further inquiries can be directed to the corresponding authors.

## Supplementary Materials

The following supporting information can be downloaded at: https://www.mdpi.com/article/10.3390/plants15081192/s1, Table S1: BSA sequencing data statistics; Table S2: BSA high-quality data statistics; Table S3: SNP/INDEL annotation result statistics of BSA-Seq; Table S4: Raw data of RNA-Seq; Table S5: RNA-Seq Map statistics; Table S6: Candidate gene expression quantity; Table S7: Primers used for quantitative real time PCR analysis; Figure S1: Distribution of SNPs on chromosomes by BSA-seq analysis Editing by Norbert Imreh.
