# Supplementary material for: Identifying Candidate Genes for Cotton Fruit Branch Length Using BSA-Seq and RNA-Seq
Source: Plants (Basel). 2026 Apr 13;15(8):1192. doi: 10.3390/plants15081192 (PMC13119465; doi:10.3390/plants15081192)
Supplement: Supplementary file 1 [file plants-15-01192-s001.zip › plants-4214142-supplementary.pdf]

Table S1. BSA sequencing data statistics

Sample	Reads_Num	Total_Bases(bp)	N(%)	GC(%)	Q20(%)	Q30(%)
P14	433583046	65471039946	0	35.59	99.18	96.98
P16	463008272	69914249072	0	35.29	99.37	97.47
SB	682300986	103027000000	0	35.42	99.09	96.76
LB	552478802	83424299102	0	35.58	99.15	96.87

Table S2. BSA high-quality data statistics

Sample	HQ_Reads	HQ_Reads(%)	HQ_Data(bp)	HQ_Data(%)
P14	423414894	0.9765	63571287695	0.971
P16	453489002	0.9794	68140548933	0.9746
SB	664593250	0.974	99817936184	0.9688
LB	539058154	0.9757	80949282571	0.9703

Table S3. SNP/INDEL annotation result statistics of BSA-Seq

	SNP		INDEL	
Type	Number	Percentage	Number	Percentage
exonic total	49996	1.39	2005	0.36
synonymous SNV	17516	0.49	-	-
nonsynonymous SNV	31306	0.87	-	-
frameshift deletion	-	-	680	0.12
frameshift insertion	-	-	454	0.08
nonframeshift deletion	-	-	441	0.08
nonframeshift insertion	-	-	370	0.07
nonframeshift substitution	-	-	0	0
stopgain	687	0.02	31	0.01
stoploss	200	0.01	14	0
unknown	287	0.01	15	0
splicing	208	0.01	96	0.02
ncRNA total	0	0	0	0
ncRNA_exonic	0	0	0	0
ncRNA_exonic; splicing	-	-	0	0
ncRNA_splicing	0	0	0	0
ncRNA_intronic	0	0	0	0
ncRNA_UTR5	-	-	0	0
intronic	147399	4.10	33959	6.05
intergenic	3102238	86.21	450496	80.23
UTR5	0	0	2	0
UTR3	0	0	0	0
UTR5; UTR3	0	0	0	0
upstream	161357	4.48	40979	7.3
downstream	128843	3.58	31821	5.67
upstream; downstream	8353	0.23	2161	0.38
Total	3598394	100	561519	100

Table S4. Disembarking data of RNA-Seq

Sample	Raw Reads No	Raw Bases(bp)	Q30 (bp)	GC (%)	N (%)	Q20 (%)	Q30 (%)
S_T0_1	37828758	5712142458	5481477540	43.08	0.003288	99.08	95.96
S_T0_2	43574366	6579729266	6311746476	43.12	0.003348	99.04	95.93
S_T0_3	39153488	5912176688	5681058849	43.1	0.001787	99.11	96.09
S_T1_1	37238572	5623024372	5394686865	42.81	0.003356	99.03	95.94
S_T1_2	50154158	7573277858	7259187071	42.84	0.003292	99.02	95.85
S_T1_3	57994912	8757231712	8401172880	42.62	0.00331	99.04	95.93
L_T0_1	42424832	6406149632	6135641222	43.49	0.003314	99.03	95.78
L_T0_2	39496684	5963999284	5725608749	43.18	0.003303	99.07	96
L_T0_3	44639498	6740564198	6454187043	43.95	0.003308	98.99	95.75
L_T1_1	38190032	5766694832	5539358773	43.37	0.001768	99.11	96.06
L_T1_2	38592858	5827521558	5579695688	43.67	0.003348	99	95.75
L_T1_3	45139750	6816102250	6565656174	43.28	0.003206	99.18	96.33

Table S5. RNA-Seq Map statistics

Sample	Clean_Reads	Total_Mapped	Multiple_Mapped	Uniquely_Mapped
S_T0_1	37406154	36018611 (96.29%)	2339254 (6.49%)	33679357 (93.51%)
S_T0_2	43040502	42218708 (98.09%)	2307014 (5.46%)	39911694 (94.54%)
S_T0_3	38710638	37983816 (98.12%)	2427534 (6.39%)	35556282 (93.61%)
S_T1_1	36781130	36190257 (98.39%)	1888651 (5.22%)	34301606 (94.78%)
S_T1_2	49540706	48555819 (98.01%)	2824557 (5.82%)	45731262 (94.18%)
S_T1_3	57315414	56235957 (98.12%)	3013178 (5.36%)	53222779 (94.64%)
L_T0_1	41935606	41311305 (98.51%)	2988739 (7.23%)	38322566 (92.77%)
L_T0_2	39066686	38563265 (98.71%)	1898026 (4.92%)	36665239 (95.08%)
L_T0_3	44065918	43420927 (98.54%)	4704084 (10.83%)	38716843 (89.17%)
L_T1_1	37787358	37233109 (98.53%)	2616102 (7.03%)	34617007 (92.97%)
L_T1_2	38093330	37535130 (98.53%)	3099802 (8.26%)	34435328 (91.74%)
L_T1_3	44685428	43984191 (98.43%)	2798287 (6.36%)	41185904 (93.64%)

Table S6 Candidate gene expression quantity

Gene_ID	GH_D02G0713	GH_D02G0744	GH_D11G0094
S_T0_1:fpkm	8.32	2.26	0.30
S_T0_2:fpkm	9.55	1.45	0.44
S_T0_3:fpkm	10.90	1.86	0.37
L_T0_1:fpkm	4.56	0.31	0.16
L_T0_2:fpkm	4.00	0.34	0.10
L_T0_3:fpkm	3.06	0.33	0.13
S_T1_1:fpkm	24.45	8.00	1.27
S_T1_2:fpkm	18.11	7.27	1.77
S_T1_3:fpkm	21.28	7.64	1.99
L_T1_1:fpkm	5.18	0.63	0.00
L_T1_2:fpkm	4.07	0.35	0.00
L_T1_3:fpkm	6.04	0.49	0.00

Table S7. Primers used for quantitative real time PCR analysis

Gene ID	Forward Primer (5' - 3')	Reverse Primer (5' - 3')
GhActin14- qPCR	ATCCTCCGTCTTGACCTTG	TGTCCGTCAGGCAACTCAT
GH_D02G0744	AACCTTACTCTCCTGATG	TGTATGTGTATGTAGAACCT
GH_D11G0094	CCTTCATTGTGGCTTCAT	TTATCGGCGGAGAACATA
GH_D02G0713	CAGTTTCTCTCAGTTTCT	GTAGTGGGTTCTTCTTAG

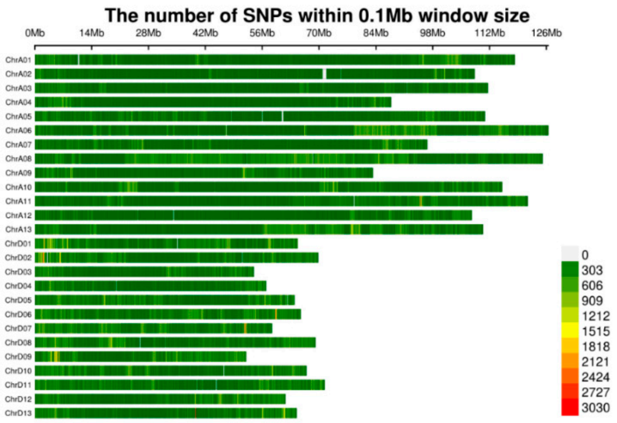

Figure S1. Distribution of SNPs on chromosomes by BSA-seq analysis Editing by Norbert Imreh.
